# Increasing the Level of IRS-1 and Insulin Pathway Sensitivity by Natural Product Carainterol A

**DOI:** 10.3390/molecules21101303

**Published:** 2016-09-29

**Authors:** Kaiqing Ma, Yanhong Miao, Yao Gao, Junsheng Tian, Li Gao, Deyong Ye, Xuemei Qin

**Affiliations:** 1 Modern Research Center for Traditional Chinese Medicine, Shanxi University, Taiyuan 030006, China; makaiqing@sxu.edu.cn(K.M.); myj15530149989@163.com (Y.M.); 18734823114@163.com (Y.G.); jstian@sxu.edu.cn (J.T.); gaoli87@sxu.edu.cn (L.G.); 2 College of Chemistry and Chemical Engineering, Shanxi University, Taiyuan 030006, China; 3 Department of Medicinal Chemistry, School of Pharmacy, Fudan University, Shanghai 201203, China

**Keywords:** carainterol A, network pharmacology, insulin receptor substrate 1, insulin signaling pathway

## Abstract

Carainterol A is a eudesmane sesquiterpenoid extracted from *Caragana intermedia*. We have reported that carainterol A showed potent glucose consumption activity in C_2_C_12_ muscle cells and the db/db mouse model. However, the mechanism of the hypoglycemic effect of carainterol A remains elusive. In this article, we present a network pharmacology approach to predict the target and signaling pathway of carainterol A which was subsequently validated in HepG2 cells. It was demonstrated that carainterol A could increase the protein levels of IRS-1 and the downstream protein kinase AKT phosphorylation at a low micromolar level. These findings suggest that carainterol A can be a valuable lead compound and a promising chemical probe for the insulin signaling pathway.

## 1. Introduction

**Figure 1:**
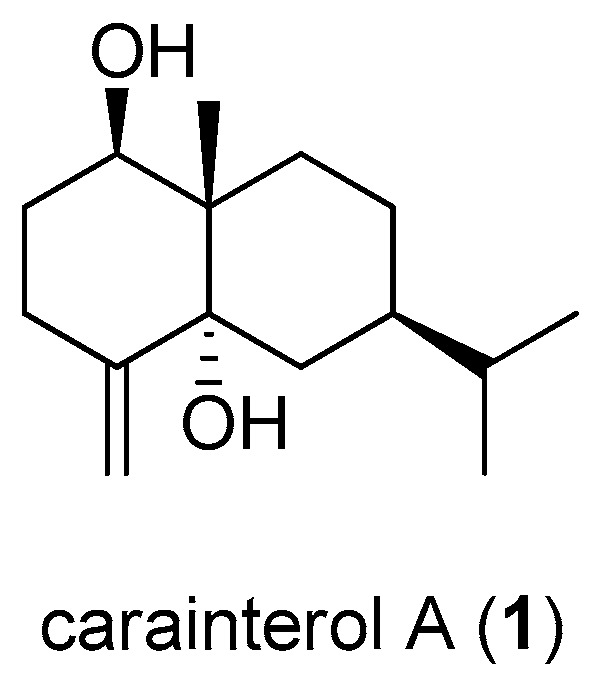
Structure of carainterol A.

Natural products and their derivatives have been an invaluable source for drug discovery [1]. Sesquiterpenoids have aroused considerable interest and attracted continuous attention as they embody impressive architectural diversity, stereochemical intricacies and pronounced biological activities. These properties continue to render sesquiterpenoids as exciting objectives for organic chemists and biologists [2]. Carainterol A (**1**), an architecturally complex and unique eudesmane sesquiterpenoid natural product (Figure 1), was isolated from the aerial part of *Caragana intermedia* which is a frequently used herb in traditional Chinese medicine. In our previous study, it is demonstrated that carainterol A showed potent activity in increasing glucose consumption in C_2_C_12_ muscle cells. Furthermore, it also displayed glucose consumption in a db/db mouse model with a MIC (minimum inhibitory concentration) value that is equivalent to that of metformin [3]. Due to its unique structure and significant biological activity, we have completed the total synthesis of carainterol A [4]. However, the pharmacological targets associated the mechanism of carainterol A remained elusive.

Identifying functional targets and clarifying the molecular mechanism of action of bioactive natural products have been proven to be particularly challenging [5]. Network pharmacology, a system biology–based methodology, has been recently employed to provide an effective mapping of the yet unexplored target space of natural products and a systematic way to extend the druggable space of proteins implicated in different complex diseases [6]. This approach has been utilized successfully for the study of the molecular mechanism of traditional Chinese medicine (TCM) [7] and single multi-targeted natural products [6,8]. For example, the PharmMapper server was used as a valuable tool for identifying targets for several natural products such as sinapic acid [9]. The mode of action by the network analysis (MANTRA) method was developed to discover the proapoptotic effect of pinosylvin [10]. Therefore, network pharmacology is supposed to be a promising approach for learning the mechanism of carainterol A.

In this study, carainterol A was analyzed using the PharmMapper and ClueGO to predict the target proteins and related pathways, respectively. Furthermore, carainterol A was evaluated in the HepG2 cells in order to clarify its biochemical action. We demonstrated that carainterol A could increase insulin pathway sensitivity through increasing the protein levels of IRS-1 and the downstream protein kinase AKT phosphorylation at a low micromolar level.

## 2. Results

### 2.1. Target and Pathway Predictions

After the removal of duplication and standardization, 118 potential molecular targets were retrieved based on the fitting score from high to low. These potential molecular targets are involved in a variety of physiological and/or pathological processes. Among the 118 protein targets, 46 targets (39%) including IGF1R, INSR, MDM2, PPARG, RXRA, PTN1, RXRB and RARG have been reported to be related to diabetes by previous studies. Sixteen pairs of compound-target interactions were found and their fitting scores of docking were greater than 3.5 (Table 1). For example, protein tyrosine phosphatase 1B (PTN1), the enzyme that catalyzes tyrosine dephosphorylation, functions as anegative regulator in both insulin and leptin signaling [11]. The mutations resulting in the loss of function of peroxisome proliferator-activated receptor γ (PPARγ) underlie the development of severe insulin resistance and overt type 2 diabetes [12]. Retinoid X receptor (RXR) agonists function as insulin sensitizers in obese mice [13]. Therefore, carainterol A may interact with these proteins and transcription factor to show potent activity in increasing glucose consumption. This is consistent with our previous findings on the pharmacological effects of carainterol A in diabetes which further argued that the PharmMapper approach was feasible and robust in the target identification of natural products.

### 2.2. Network Construction and Analysis

**Figure 2:**
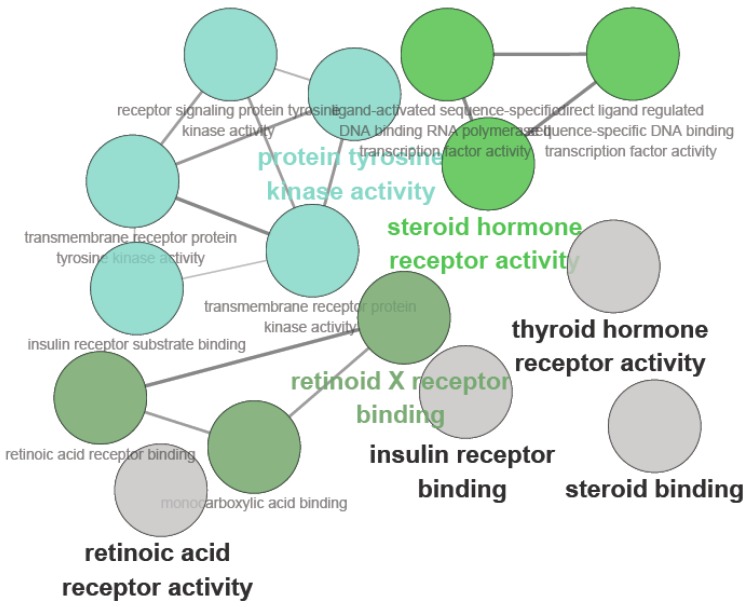
Functionally grouped network for the candidate target of carainterol A.

Bioinformatics enrichment tools have contributed greatly in the study of gene function analysis in traditional Chinese medicine [14]. To address the challenges of the functional analysis of gene lists, some preliminary studies on the high-throughput enrichment tools were developed. The fished target proteins were further used to map them to diabetes using the PharmGKB, KEGG, CTD and TTD databases and extracted for analysis. The ClueGo analysis for multi-targets of carainterol A is shown in Figure 2.

**Figure 3:**
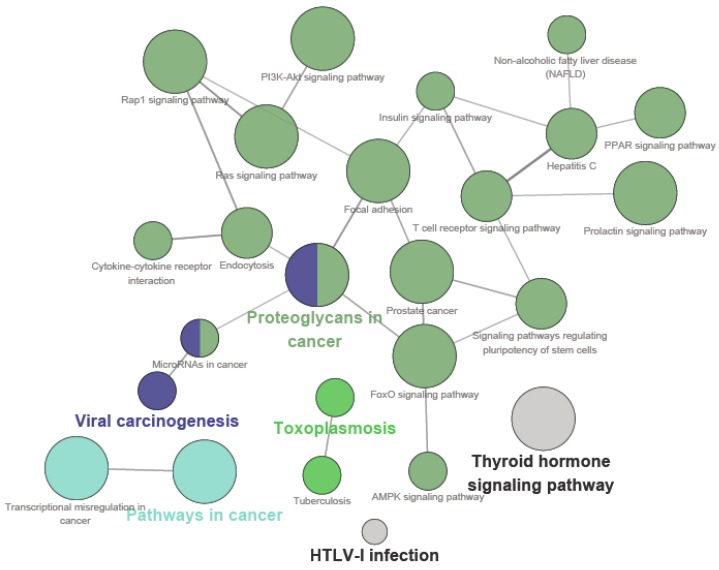
Pathway grouped network for the candidate targets of carainterol A.

The main molecular functions were divided into five categories: insulin receptor substrate binding, insulin receptor binding, protein tyrosine kinase activity, retinoid X receptor binding and steroid hormone receptor activity. The main pathways included in the ClueGo analysis were as follows: the PI3K-Akt signaling pathway, pathways in cancer, proteoglycans in cancer, the FOXO signaling pathway, the prolactin signaling pathway, the AMPK signaling pathway, the PPAR signaling pathway, the insulin signaling pathway and the thyroid hormone signaling pathway (Figure 3).

In our previous study, carainterol A showed potent glucose consumption activity in C_2_C_12_ muscle cells. Furthermore, it also displayed increasing glucose consumption in a db/db mouse model with a micromolar value that is equivalent to that of metformin. The present study indicated that carainterol A possesses multipharmacology attributes through different target groups such as insulin receptor substrate binding, insulin receptor binding and protein tyrosine kinase activity. Thyroid hormone receptor subtype-β agonists could improve glucose tolerance and insulin sensitivity in a dose-dependent manner [15]. Therefore, these could be the potential molecular mechanisms by which carainterol A improves glucose utilization. Furthermore, some new activities were found to be related with carainterol A, which might offer some new insights for the application of carainterol A. For example, the AKT signaling pathway plays a crucial role in the insulin signaling pathway. Meanwhile, the aberrant AKT signaling is also involved in many sporadic cancers as well as in several dominantly inherited cancer syndromes [16]. Therefore, the probes that have an influence in the AKT signaling might possess antitumor activity. Interestingly, several pathways involved in cancer were highlighted in the ClueGO analysis of carainterol A, such as in prostate cancer.

### 2.3. Pathway Verification

**Figure 4:**
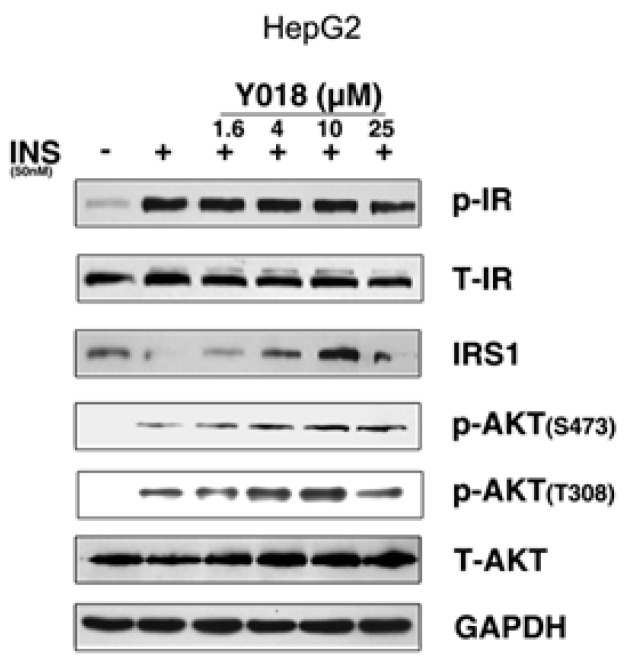

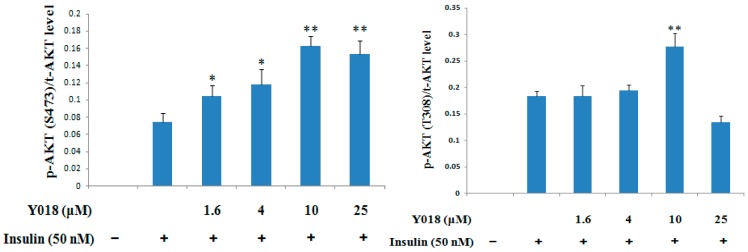
The effect of carainterol A on the insulin signaling pathway in HepG2 cells. Data are presented as means ± SD (* *p* < 0.05, ** *p* < 0.01) from three independent experiments. “+”: with insulin; “−”: without insulin.

Guided by the in silico prediction results, we explored the effects of carainterol A on the insulin signaling pathway. As shown in Figure 4, carainterol A (Y018) showed no impact on the insulin receptor phosphorylation level in HepG2 cells. However, Y018 could significantly increase the protein levels of IRS-1 at 4 μmol in a concentration-dependent manner. Moreover, Y018 could increase downstream protein kinase AKT phosphorylation including the S473 and T308 phosphorylation sites.

**Figure 5:**
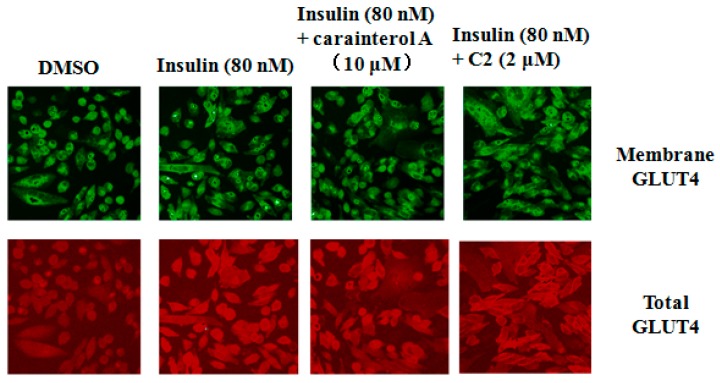
GLUT4 membrane translocation.

IRS-1 has been implicated in insulin signaling transduction from the insulin receptor to AKT phosphorylation, followed by GLUT4 translocation [17]. To further explore the molecular mechanism of carainterol A, GLUT4 translocation was evaluated to determine whether carainterol A affected the downstream signal. The results indicated that Y018 unexpectedly did not increase the insulin-stimulated human GLUT4 on the membrane at the concentration tested, which was 10 μM, while the positive compound C2, a known PTP1B selective inhibitor [18], could significantly increase the amount of GLUT4 on the membrane (Figure 5).

## 3. Discussion

Elucidation of the pharmacological profile of a single natural product from natural medicine has been the task for many researchers over the decades. Compared to structural optimization for inhibiting a specific target, multi-target drug approaches were employed recently to study the multi-target character of a single natural product [19]. Among these approaches, network pharmacology was utilized extensively to provide a systemic understanding of the molecular mechanism of natural products. In the current study, the PharmMapper server with pharmacophore reverse screening technology was employed to investigate and predict the potential anti-diabetic targets of carainterol A. The pathway enrichment was analyzed using ClueGO software and the results suggested that carainterol A might have an influence on the insulin signaling pathway and the cancer-related pathway. Eventually, the insulin signaling pathway was selected for experimental validation in order to explore the useful clues for the further study of the molecular mechanism of action. The results indicated that carainterol A could significantly increase the protein levels of IRS-1 at 4 μmol.

The dysregulation of IRS-1 has been implicated in the pathogenesis of type II diabetes and cancer [20]. Thus, it was proposed that therapeutic interventions to mediate the IRS-1 protein level might be a novel approach for the treatment of insulin resistance [21,22]. Recently, it was reported that delayed IRS-1 degradation by the TBC1D3 gene could promote insulin signaling [23]. Moreover, total saponins [24] and Chinese herbal medicine [25,26] had been demonstrated to show anti-diabetic activity through up-regulating the protein expression of IRS-1. To the best of our knowledge, carainterol A is the first natural product that has been reported to increase insulin pathway sensitivity based on the regulation of the IRS-1 level.

Although no influence on GLUT4 translocation was observed, it was proposed that carainterol A may increase glucose consumption through the unknown downstream signaling component of IRS-1. Our results have implied that carainterol A could serve as a valuable chemical probe for the insulin signaling pathway investigation.

## 4. Materials and Methods

### 4.1. Target Prediction by PharmMapper

PharmMapper, a web server for potential drug target identification, is based on the use of a pharmacophore mapping approach [27]. The best mapping poses of the query molecule against all the pharmacophore models in PharmTargetDB is automatically found. The top N best-fitted hits with appropriate target annotations and the aligned poses of the respective molecules are listed. The molecular file of carainterol A was downloaded from the PubChem database (CID: 1548943) and uploaded to the PharmMapper server. The search started using the maximum generated conformations at 300 by selecting “all targets (7302)” option and default value of 300 for the number of reserved matched targets. The default settings were used for other parameters. Their structures were put into the PharmMapper database [28] for target prediction.

In this study, all targets retrieved from the above were sent to database UniProt [29] for target name standardization, which were further subjected to PharmGkb [30]. Therapeutic target database and the comparative toxicogenomics database were employed to eliminate the noise, errors and overlaps to ensure the quality of target database. Afterward, the fished target proteins were further used to map to diabetes using the PharmGKB, KEGG, CTD and TTD databases. Then, the potential targets related to diabetes were extracted from the Therapeutic Target (TTD), DrugBank, and PharmGkb databases.

### 4.2. Functional and Pathway Analysis by ClueGO

ClueGO, a Cytoscape plug-in, is a professional software to enhance the biological interpretation and to analyze the functionally grouped terms in the form of networks and graphs [31]. It was used to perform functional and pathway analysis for the targets related to carainterol A. Simple text version of the targets in gene identifiers type was directly uploaded into the ClueGO software (Institute for Genomics and Bioinformatics Graz University of Technology, Graz, Austria). Based on the hypergeometric distribution, enrichment and depletion tests were employed for terms and groups as two-sided (enrichment/depletion) tests. The network type was adopted as a “Medium” network. In order to develop the annotations network, functional groups were visualized in the network using ClueGO which employed the organic layout algorithm.

### 4.3. The Experiments of Carainterol a (Y018) in Insulin-Sensitizing

The protocol of the pathway verification experiment refers to the paper published by Shen et al. [17]. Y018 was synthesized through the method in our previous study [4].

HepG2 cells were incubated in 12-well plates overnight and the starved cells for 4 h (maintained compound concentration). Then, the cell was stimulated for 5 min by the insulin and cells were collected for the Western blot experiments. In the experiments of the regulation of protein level of IRS-1, the compounds were tested after incubation in the serum.

### 4.4. The Influence of Y018 on Membrane Translocation of GLUT4

In the plasmid pEGFPN1-rGLUT4-myc, c-myc epitope sequence was inserted into the GLUT4 protein extracellular region of rat and the C-terminus was coupled with EGFP green fluorescent protein form a fusion protein. The plasmid was stably transfected into CHO-K1 cells. In the experiment, in the case of impermeable membrane, anti-c-myc antibody as well as the red fluorescent secondary antibodies was used for indirect immunostaining. Green fluorescence intensity indicated the total GLUT4 in the cells and red fluorescence intensity indicated the total GLUT4 on the cell membrane.

## 5. Conclusions

We used PharmMapper and ClueGO software to predict the main disease-related targets and pathways of carainterol A. Furthermore, the effect on the insulin signaling pathway was validated as the main biological mechanism of carainterol A through increasing the protein level of IRS-1 and the downstream protein kinase AKT phosphorylation level. Further studies towards how exactly carainterol A regulates the protein level of IRS-1 are in progress and will be reported in due course.

## Figures and Tables

**Table 1:** The fitting score of docking of carainterol A with potential targets.

No.	Target Gene	Uniprot ID	Protein Name	Fit	Norm Fit
1	GSTA1	P08263	Glutathione S-transferase A1	4.341	0.5426
2	RXRA	P19793	Retinoic acid receptor RXR-α	3.916	0.5594
3	SULT2A1	Q06520	Bile salt sulfotransferase	3.77	0.7541
4	RARG	P13631	Retinoic acid receptor γ	3.753	0.4691
5	SRC	P12931	Proto-oncogene tyrosine-protein kinase Src	3.725	0.4139
6	PTN1	P18031	Tyrosine-protein phosphatase non-receptor type 1	3.725	0.4139
7	NR1I3	Q14994	Nuclear receptor subfamily 1 group I member 3	3.72	0.6201
8	SHBG	P04278	Sex hormone-binding globulin	3.711	0.4638
9	GSTP1	P09211	Glutathione S-transferase P	3.671	0.5244
10	MAPK14	Q16539	Mitogen-activated protein kinase 14	3.657	0.3657
11	NR3C1	P04150	Glucocorticoid receptor	3.637	0.3306
12	KIT	P10721	Mast/stem cell growth factor receptor	3.628	0.4031
13	TTPA	P49638	Alpha-tocopherol transfer protein	3.591	0.2993
14	PLA2G2A	P14555	Phospholipase A2, membrane associated	3.586	0.4482
15	PPARG	P37231	Peroxisome proliferator-activated receptor γ	3.549	0.507
16	VDR	P11473	Vitamin D3 receptor	3.51	0.27
